# The impact of rosuvastatin on hypothalamic–pituitary–testicular axis activity in metformin-treated and metformin-naïve men with low testosterone levels: a pilot study

**DOI:** 10.1007/s43440-021-00289-1

**Published:** 2021-06-04

**Authors:** Robert Krysiak, Marcin Basiak, Witold Szkróbka, Bogusław Okopień

**Affiliations:** Department of Internal Medicine and Clinical Pharmacology, Medical University of Silesia, Medyków 18, 40-752 Katowice, Poland

**Keywords:** Androgens, Coronary artery disease, Metformin, Statins

## Abstract

**Background:**

Intense statin therapy was found to impair testosterone production in men. Metformin administered to subjects with hypergonadotropic hypogonadism decreased gonadotropin production. The current study was aimed at investigating whether metformin treatment modulates the impact of high-dose rosuvastatin therapy on hypothalamic–pituitary–testicular axis activity in men.

**Methods:**

The study included 43 very high cardiovascular risk men with late-onset hypogonadism, 20 of whom had been treated with metformin (1.7–3 g daily) for at least 6 months. In all subjects, unsuccessful initial statin treatment was replaced with rosuvastatin (20–40 mg daily). Plasma lipid levels, glucose homeostasis markers, as well as circulating levels of gonadotropins, testosterone, bioavailable testosterone, dehydroepiandrosterone-sulfate, prolactin, estradiol and creatinine were measured at the beginning of the study and 4 months later in 28 individuals in whom rosuvastatin reduced LDL cholesterol levels to below 70 mg/dL.

**Results:**

There were no differences between treatment-induced changes in plasma lipids. In both study groups, rosuvastatin reduced total and bioavailable testosterone levels. However, only in metformin-naïve men, rosuvastatin increased LH and FSH levels and slightly impaired insulin sensitivity. The impact on gonadotropin concentrations correlated with treatment-induced decrease in testosterone levels. There were no significant differences between baseline and posttreatment values of dehydroepiandrosterone-sulfate, prolactin, estradiol and the glomerular filtration rate.

**Conclusion:**

The obtained results suggest that metformin prevents the compensatory increase in gonadotrope function induced by intense statin therapy.

## Introduction

Most steroid hormones are synthetized from cholesterol contained in low-density lipoprotein (LDL) particles, which are uptaken by the adrenals and gonads and used as a substrate for steroidogenesis [1]. Therefore, disorders or other states associated with very low levels of low-density lipoprotein (LDL) cholesterol may impair production of adrenal and gonadal hormones [1]. A marked decrease in LDL-cholesterol is induced by 3-hydroxy–3-methylglutaryl-CoA (HMG-CoA) reductase inhibitors (statins), one of the most commonly prescribed drugs in the primary and secondary prevention of cardiovascular disease [2]. A meta-analysis of randomized controlled studies showed that statins slightly decrease circulating testosterone levels in both men and women [3]. The impact on androgens was more pronounced for testicular than adrenal hormones, observed mainly in patients receiving intense statin therapy and correlated with the reduction in total testosterone levels [4, 5].

The reproductive axis is modulated also by metformin, which is the preferred first-line agent in patients with type 2 diabetes mellitus and other insulin resistant states [6]. Metformin administered at high doses to postmenopausal women reduced follicle-stimulating hormone (FSH) concentrations, as well as tended to decrease luteinizing hormone (LH) levels [7]. In men with primary hypogonadism, the drug decreased both FSH and LH concentrations [8]. In women with polycystic ovary syndrome, metformin decreased both LH levels and the LH/FSH ratio [9, 10]. Finally, metformin reduced LH and FSH secretion induced in animals by administration of gonadotropin-releasing hormone or activin [11]. This effect was probably mediated by 5ʹ-adenosine monophosphate-activated protein kinase (AMPK) signaling pathway [11], which is a sensor of energy status that maintains cellular energy homeostasis [12]. Unlike gonadotropins, the impact of metformin on testosterone in men differed between studies: the drug either reduced [13] or had a neutral effect [14] on testosterone levels.

To the best of our knowledge, no previous study has assessed the impact of metformin/statin combination on androgen levels in men. In women with polycystic ovary syndrome, metformin administered together with rosuvastatin was superior to metformin alone in reducing free and total testosterone levels [15]. Similarly, the impact of metformin/simvastatin combination therapy on testosterone levels in women with this disorder was stronger than the effect of both drugs administered alone [16]. Finally, simvastatin reduced plasma levels of testosterone, free testosterone, androstenedione and dehydroepiandrosterone-sulfate (DHEA-S) and tended to reduce 17-hydroxyprogesterone in metformin-treated women with non-classic congenital adrenal hyperplasia, but in metformin-treated women with normal androgen levels [17]. The paucity of data, as well as common use of metformin and a HMG-CoA reductase inhibitor by the same patient encouraged us to investigate whether metformin treatment modulates the impact of high-dose rosuvastatin therapy on hypothalamic–pituitary–testicular axis activity in men with late-onset hypogonadism.

## Materials and methods

The study was approved by the institutional review board and adhered to the 1964 declaration of Helsinki and its later amendments. All participants gave written informed consent after having received detailed information about potential benefits and risks as well as alternative treatment options, and after receiving a complete description of the study.

### Patients

The participants (*n* = 43) of this non-randomized, uncontrolled pilot study were recruited among men (40–75 years) at very high cardiovascular risk. To be included, they were required to have plasma LDL cholesterol levels in the range between 70 and 130 mg/dL, despite compliance with a lipid-lowering diet and statin treatment, as well as low testosterone levels, defined as total testosterone concentration below 10.4 nmol/L (3.0 ng/mL).

The study population consisted of two groups. Group A (*n* = 20) was selected among subjects treated for at least 6 months with metformin (1.7–3 g daily) because of impaired fasting glucose, impaired glucose tolerance or other insulin resistance states. In turn, group B included 23 men not receiving hypoglycemic therapy. Power analysis was not be performed because of the pilot nature of the study and a lack of data in the literature required for sample size calculation. To minimize the effect of seasonal fluctuations in the measured variables, similar proportions of participants were recruited between December and February (51%), and between June and August (49%).

Potential participants were excluded if they met any of the following criteria: unstable coronary artery disease, myocardial infarction or stroke within 3 months preceding the study, symptomatic congestive heart failure, chronic inflammatory or autoimmune disorders, diabetes or any other endocrine disorder, kidney or liver failure, malabsorption syndrome, statin or metformin intolerance, as well as poor patient compliance. We also excluded patients treated for 4 months preceding the study with rosuvastatin, glucocorticoids, other drugs modulating hypothalamic–pituitary–gonadal axis and/or hypothalamic–pituitary–adrenal axis activities, or with drugs known to interact with statins or metformin.

### Study design

Previous statin treatment was replaced with rosuvastatin, administered at the daily dose of 20 mg once daily at bedtime. After 6 weeks of therapy, LDL cholesterol levels were again measured and if they were still above 70 mg/dL, the dose of rosuvastatin was increased to 40 mg daily. Over the entire study period (4 months), previously treated subjects received the same daily dose of the remaining drugs (including metformin in group B) as before enrollment. The participants were requested to swallow the tablets whole with a drink of water and do not crush, chew, or break it. Metformin was swallowed with or immediately after meals to minimize potential adverse effects. All men were also recommended: (a) to limit cholesterol intake to less than 200 mg per day, total fat intake to less than 30% of energy consumed and saturated fat intake to less than 7% of total energy intake, (b) to increase fiber intake to 15 g per 1000 kcal, as well as (c) do at least 150 min of moderate intensity activity every week. Medication adherence was measured fortnightly by pill count and by means of Morisky–Green test. Compliance with non-pharmacological recommendations was assessed by analysis of individual dietary questionnaires and of diaries in which the participants continuously recorded all their activities.

### Laboratory assays

Venous blood samples were taken 12 h after the last meal in a quiet, temperature-controlled room (24–25 °C) in constant daily hours (between 7.00 and 8.00 a.m.). Before venipuncture, the participants had been resting in the seated position for a minimum 30 min. We also retrospectively analyzed concentrations of anti-Müllerian hormone in stored serum samples of 10 subjects in each treatment group. All laboratory assays were carried out by technicians who were blinded to the treatment group assignments. Measurements were performed in duplicate within a single analytical session, and final results were averaged. Serum levels of insulin, DHEA-S, total testosterone, estradiol, sex-hormone binding globulin, anti-Müllerian hormone and prolactin were assayed by direct chemiluminescence using acridinium ester technology (ADVIA Centaur XP Immunoassay System, Siemens Healthcare Diagnostics, Munich, Germany). Plasma levels of total cholesterol, LDL cholesterol, high-density lipoprotein (HDL) cholesterol, and triglycerides, glucose and creatinine were assayed by routine techniques using commercially available kits. Bioavailable testosterone was estimated from total testosterone and sex hormone-binding globulin with the Vermeulen formula, using the online calculator (www.issam.ch/freetesto.htm). The homeostatic model assessment 1 of insulin resistance (HOMA1-IR) was calculated from fasting plasma glucose and insulin concentration according to the formula described by Matthews et al. [18], while the estimated glomerular filtration rate was calculated using the Modification of Diet in Renal Disease study equation.

### Statistical analysis

The outcome variables were log-transformed to mitigate the effects of the non-normal distributions, as well as were transformed back for reporting in the tables. Comparisons between both groups and between percent changes from baseline after adjustment for baseline values were performed with Student’s *t* test for independent samples. Comparisons between baseline and follow-up values within each group were carried out using paired Student *t* test. Categorical variables were compared using the *χ*^2^ test. The Pearson correlation coefficient was employed to determine the correlation between the variables. The data were analyzed with a predetermined level of significance set to *p* value corrected for multiple testing below 0.05.

## Results

At study entry, there were no differences between groups A and B in age, smoking, body mass index, systolic and diastolic blood pressure, circulating levels of total cholesterol, LDL cholesterol, HDL cholesterol, triglycerides, glucose, FSH, LH, total testosterone, bioavailable testosterone, DHEA-S estradiol, prolactin and anti-Müllerian hormone, as well as in HOMA1-IR and the glomerular filtration rate (Tables 1, 2, and 3).

**Table 1:** Baseline characteristics of patients

Variable	Group A^a^	Group B^b^	*p* value (Group A vs. Group B)
Number (n)	14	14	–
Age (years; mean [SD])	58 (10)	59 (10)	0.7934
Smokers (%)	50	43	–
Body mass index (kg/m^2^; mean [SD])	28.2 (4.3)	29.3 (4.8)	0.5286
Systolic blood pressure (mmHg; mean [SD])	140 (16)	143 (18)	0.6450
Diastolic blood pressure (mmHg; mean [SD])	91 (8)	92 (10)	0.7725

Only data of patients who achieved LDL cholesterol levels below 70 mg/dL at the end of the treatment period were included in the final analyses

^a^Metformin-treated men

^b^Metformin-naive men

**Table 2:** Effect of rosuvastatin on plasma lipids, glucose homeostasis markers and the glomerular filtration rate in metformin-treated and metformin-naïve men with low testosterone levels

Variable	Group A^a^	Group B^b^	*p* value (Group A vs. Group B)
Total cholesterol (mg/dL; mean [SD])			
Baseline values	178 (31)	184 (29)	0.6014
Follow-up values	137 (21)	141 (25)	0.6505
*p* value (follow-up vs. baseline values)	**0.0004**	**0.0003**	–
LDL-cholesterol (mg/dL; mean [SD])			
Baseline values	96 (15)	98 (16)	0.7352
Follow-up values	56 (9)	58 (8)	0.5397
*p* value (follow-up vs. baseline values)	** < 0.0001**	** < 0.0001**	–
HDL-cholesterol (mg/dL; mean [SD])			
Baseline values	44 (10)	47 (11)	0.4570
Follow-up values	49 (8)	48 (8)	0.7435
*p* value (follow-up vs. baseline values)	0.1560	0.7854	–
Triglycerides (mg/dL; mean [SD])			
Baseline values	175 (48)	184 (51)	0.6347
Follow-up values	148 (37)	156 (46)	0.6164
*p* value (follow-up vs. baseline values)	0.1075	0.1392	–
Glucose (mg/dl; mean [SD])			
Baseline values	100 (11)	95 (12)	0.1796
Follow-up values	98 (10)	98 (13)	1.0000
*p* value (follow-up vs. baseline values)	0.6189	0.5312	–
HOMA1-IR (mean [SD])			
Baseline values	3.7 (1.1)	3.2 (0.9)	0.1996
Follow-up values	3.5 (1.0)	4.0 (1.1)*	0.2194
*p* value (follow-up vs. baseline values)	0.6189	**0.0450**	–
Estimated glomerular filtration rate (ml/min/1.73m^2^; mean [SD])			
Baseline values	83 (14)	85 (15)	0.7183
Follow-up values	87 (17)	88 (14)	0.8664
*p* value (follow-up vs. baseline values)	0.5028	0.5890	–

Only data of patients who achieved LDL cholesterol levels below 70 mg/dL at the end of the treatment period were included in the final analyses. Statistically significant results are marked in bold

*Percent changes from baseline after adjustment for baseline values more pronounced than in group A

^a^Metformin-treated men

^b^Metformin-naive men

**Table 3:** Effect of rosuvastatin on the investigated hormones in metformin-treated and metformin-naïve men with low testosterone levels

Variable	Group A^a^	Group B^b^	*p* value [Group A vs. Group B]
FSH (IU/L; mean [SD]	7.8 (2.5)	7.1 (2.0)	0.4207
Baseline values	7.6 (2.3)	9.7 (2.2)*	**0.0204**
Follow-up values	0.8274	**0.0030**	–
*p* value (follow-up vs. baseline values)			
LH (IU/L; mean [SD]			
At the beginning of the study	6.5 (1.8)	5.9 (1.6)	0.3598
At the end of the study	6.0 (1.5)	7.4 (2.0)*	**0.0460**
*p* value (post-treatment vs. baseline)	0.4318	**0.0376**	–
Total testosterone (nmol/L; mean [SD])			
Baseline values	8.0 (1.4)	8.3 (1.2)	0.5480
Follow-up values	6.8 (1.6)	6.7 (1.8)	0.8777
*p* value (follow-up vs. baseline values)	**0.0445**	**0.0103**	–
Calculated bioavailable testosterone (nmol/L; mean [SD])			
Baseline values	3.1 (0.6)	3.2 (0.6)	0.6629
Follow-up values	2.5 (0.7)	2.7 (0.6)	0.4243
*p* value (follow-up vs. baseline values)	**0.0221**	**0.0365**	–
DHEA-S (µmol/L; mean [SD])			
Baseline values	3.8 (0.9)	3.6 (1.4)	0.6567
Follow-up values	4.0 (1.0)	3.5 (1.1)	0.2194
*p* value (follow-up vs. baseline values)	0.5828	0.8352	
Estradiol (pmol/L; mean [SD])			
Baseline values	120 (42)	132 (46)	0.4774
Follow-up values	110 (37)	124 (34)	0.3068
*p* value (follow-up vs. baseline values)	0.5097	0.6052	–
Prolactin (ng/mL)			
Baseline values	10.8 (4.0)	11.4 (4.7)	0.7190
Follow-up values	10.4 (3.8)	11.0 (5.2)	0.7302
*p* value (follow-up vs. baseline values)	0.7883	0.8326	–
Anti-Müllerian hormone (pmol/L; mean [SD])^c^			
Baseline values	34.5 (12.8)	29.5 (11.3)	0.3667
Follow-up values	36.0 (14.2)	42.2 (12.0)*	0.3056
*p* value (follow-up vs. baseline values)	0.8069	**0.0254**	–

Only data of patients who achieved LDL cholesterol levels below 70 mg/dL at the end of the treatment period were included in the final analyses. Statistically significant results are marked in bold

*Percent changes from baseline after adjustment for baseline values more pronounced than in group A

^a^Metformin-treated men

^b^Metformin-naive men

^c^Retrospective analysis of samples of 10 men from each group

Rosuvastatin treatment was well tolerated. Two patients from group B prematurely terminated the study owing to vomiting and diarrhea, while one patient from group A discontinued treatment because of muscle pain and weakness. At the end of the study, LDL cholesterol levels below 70 mg/dL were achieved by 28 patients (14 subjects in each treatment group), and only data of these patients were included in the final analyses. The mean dose of metformin in group A was 2.38 (0.55) g daily. The mean daily doses of rosuvastatin were similar in both groups [group A: 31.4 (10.2) mg; group B: 30.0 (10.4) mg; *p* = 0.7221].

In both study groups, rosuvastatin reduced total cholesterol and LDL cholesterol, as well as total and bioavailable testosterone. In group B, but not in group A, rosuvastatin increased levels of LH, FSH and anti-Müllerian hormone, as well as HOMA1-IR. There were no significant differences between baseline and posttreatment values of DHEA-S, estradiol, prolactin and the glomerular filtration rate. At the end of the study, both groups differed in FSH and LH levels (Tables 2 and 3).

The impact of rosuvastatin on total testosterone inversely correlated with baseline total testosterone levels [group A: *r* = − 0.43 (*p* = 0.0004); group B: *r* = − 0.39 (*p* = 0.0025)], as well as positively correlated with treatment-induced changes in total cholesterol [group A: *r* = 0.32 (*p* = 0.0124); group B: *r* = 0.35 (*p* = 0.0098)] and LDL cholesterol [group A: *r* = 0.38 (*p* = 0.0037); group B: *r* = 0.41 (*p* = 0.0006)]. Similarly, the reduction in bioavailable testosterone inversely correlated with its baseline values [group A: *r* = − 0.45 (*p* = 0.0002); group B: *r* = − 0.42 (*p* = 0.0006)], as well as positively correlated with the changes in total cholesterol [group A: *r* = 0.37 (*p* = 0.0071); group B: *r* = 0.44 (*p* = 0.0004)] and LDL cholesterol [group A: *r* = 0.39 (*p* = 0.0011); group B: *r* = 0.48 (*p* = 0.0001)]. In group B, but not in group A, there were correlations between treatment-induced changes in testosterone and gonadotropins [*r* = 0.46 (*p* = 0.0002) between Δtotal testosterone and ΔLH; *r* = 0.29 (*p* = 0.0234) between Δtotal testosterone and ΔFSH; *r* = 0.51 (*p* < 0.0001) between Δbioavailable testosterone and ΔLH; and *r* = 0.34 (*p* < 0.0185) between Δbioavailable testosterone and ΔFSH]. The remaining correlations were weak and not statistically significant.

## Discussion

The current study provides some arguments in favor of benefits of statin/metformin combination therapy. Firstly, this combination therapy was well tolerated and rarely had to be discontinued. Secondly, metformin prevented statin-induced worsening of insulin sensitivity in subjects with late-onset hypogonadism and prediabetes, constituting a population particularly prone to the development of diabetes [19]. Finally, concomitant metformin treatment normalized secretory function of gonadotropes, which were stimulated by low concentrations of circulating testosterone. However, only in metformin-naïve men, rosuvastatin exerted a stimulatory effect on secretory function of Sertoli cells. The neutral effect on anti-Müllerian hormone may be undesirable in men wanting to farther children, because Sertoli cells are known to nourish the developing sperm cells throughout the process of spermatogenesis [20].

High-dose rosuvastatin treatment resulted in a decrease in total and bioavailable testosterone levels in both groups of patients with late-onset hypogonadism and this effect may be explained by the fact that rosuvastatin action limited the amount of cholesterol for steroidogenesis in Leydig cells. Taking into account that rosuvastatin-induced decrease in testosterone inversely correlated with their baseline values, it seems that the impact on testosterone secretion depends on the severity of hypogonadism and is particularly evident in its most severe forms. Because of conflicting results in the literature, the clinical significance of this finding is difficult to interpret. Although low testosterone levels correlate with increased cardiovascular morbidity and mortality [21, 22], administration of exogenous testosterone was found to increase cardiovascular risk in men [23, 24]. Therefore, it remains unexplained whether low androgen concentrations contribute to cardiovascular disease or are only a marker of poor general health [25]. If the former explanation is correct, treatment-induced decrease in testosterone levels may attenuate numerous benefits of statin therapy. Theoretically, simultaneous administration of exogenous testosterone may restore these benefits and this issue will be verified in our future studies. The unfavorable effect on testosterone levels may be more pronounced in individuals with very low post-treatment levels of LDL cholesterol. In line with this explanation, abetalipoproteinemia, a disorder characterized by very low levels of LDL and other apolipoprotein B-containing lipoproteins, makes subjects susceptible to primary hypogonadism and chronic adrenal failure [26].

Although, rosuvastatin decreased mean total and bioavailable testosterone levels in both treatment groups, there were between-group differences in the impact of rosuvastatin on gonadotropin levels. Due to the observational nature, the current study does not identify mechanisms underlying this finding. Similar posttreatment values of total and calculated bioavailable testosterone in both treatment arms suggest that metformin does not modulate rosuvastatin action at the level of Leydig cells. Taking into account that in metformin-naïve subjects, the degree of testosterone reduction correlated with an increase in gonadotropin (particularly LH) levels, the most probable explanation of the obtained results is a modulatory effect of metformin at the level of the hypothalamus and/or pituitary. Metformin increases AMPK activity in these structures [27], as well as was found to enhance the endogenous hypothalamic dopaminergic tone [28]. Moreover, gonadotropes are a population of pituitary cells with the most abundant expression of AMPK and this kinase mediates the impact of metformin on gonadotropin secretion by cultures of primary pituitary cells [11]. Both these mechanisms also well explain why gonadotropin-lowering effects of metformin correlated with the degree of improvement in insulin sensitivity [7, 8]. In line with this explanation, testosterone increased dopamine content in cultures of hypothalamic cells [29], whereas dopamine content in the anterior pituitary lobe was lower in castrated than intact animals [30]. Another evidence supporting the correctness of this explanation is dimorphism in the effect of rosuvastatin on anti-Müllerian hormone. This hormone is produced by the Sertoli cells of the testes and its secretion is regulated by FSH [31]. Rosuvastatin increased anti-Müllerian hormone levels in metformin-naïve subjects and this effect correlated with the increase in circulating FSH levels. In turn, concomitant treatment with metformin prevented the impact of rosuvastatin on both FSH and anti-Müllerian hormone.

Theoretically, the modulatory effect of metformin might have been secondary to the conversion of aromatable androgens to estrogens. Estrogens play and important role in male reproduction and the negative regulatory gonadotropin feedback [32], stimulate hypothalamic AMPK activity [33], as well as were found to increase dopamine turnover in hypothalamic neuroendocrine dopaminergic neurons [34]. Our results, however, do not support such an explanation. Estradiol levels did not differ between the groups, were at a similar level at the beginning and at the end of the study, as well as did not correlate with the impact of treatment on gonadotropins and testosterone.

The participants of the present study received aggressive statin therapy (rosuvastatin at the daily dose of 20–40 mg) and a moderate or high dose of metformin (1.7–3 g daily). Previous observations showed that metformin administered in combination with simvastatin or atorvastatin had a beneficial effect on glucose homeostasis [35]. The most favorable effect (a reduction in glucose levels) was observed if subjects received small doses of both metformin and a statin. In turn, the impact of metformin plus a moderate dose of simvastatin was limited to a small improvement in insulin resistance. A neutral effect on glucose homeostasis of rosuvastatin–metformin combination therapy, contrasting with the unfavorable effect of rosuvastatin monotherapy on HOMA1-IR, probably resulted from the reversal by metformin of metabolic consequences of potent inhibition of HMG-CoA reductase activity. This finding is an argument in favor of concomitant metformin treatment in rosuvastatin-treated men at high risk for diabetes.

The current study has several important methodological limitations that should be considered when interpreting our findings. The main limitation is that our study was a small-scale, preliminary study, and therefore, the obtained results should be confirmed in a randomized controlled trial. The current study did not investigate hard clinical endpoints, including morbidity and mortality rates. The study design does not allow to totally exclude the “regression toward the mean” effect, occurring when an extreme variable on the first measurement is closer to the average on subsequent measurements [36]. It cannot also be also ruled out that the heterogeneity of previous statin therapy might have had an impact on the obtained results. It is uncertain whether the obtained results reflect a “class effect” of HMG-CoA reductase inhibitors or represent a specific effect of rosuvastatin. Finally, because both study groups were characterized by different glycemic profiles, it would be interesting to compare head-to-head endocrinological effects of statin/metformin combination therapy and statin monotherapy in prediabetic patients without the immediate necessity of metformin treatment.

Summing up, rosuvastatin reduced total and bioavailable testosterone levels in both metformin-naïve and metformin-treated men with hypogonadism. However, only in metformin-naïve men, rosuvastatin increased gonadotropin levels and this effect correlated with the degree of reduction in testosterone levels. The obtained results suggest that metformin prevents the compensatory increase in gonadotrope function induced by intense statin therapy. Because of numerous study limitations, the obtained results should be interpreted with caution and have to be verified in future studies.

## Abbreviations

AMPK: 5ʹ-adenosine monophosphate-activated protein kinase
DHEA-S: Dehydroepiandrosterone-sulfate
FSH: Follicle-stimulating hormone
HDL: High-density lipoprotein
HMG-CoA: 3-hydroxy–3-methylglutaryl coenzyme A
HOMA1-IR: Homeostasis model assessment 1 of insulin resistance index
LH: Luteinizing hormone
LDL: Low-density lipoprotein
SD: Standard deviation
